# Prognostic Significance of Deregulated Dicer Expression in Breast Cancer

**DOI:** 10.1371/journal.pone.0083724

**Published:** 2013-12-30

**Authors:** Emer Caffrey, Helen Ingoldsby, Deirdre Wall, Mark Webber, Kate Dinneen, Laura S. Murillo, Celine Inderhaug, John Newell, Sanjeev Gupta, Grace Callagy

**Affiliations:** 1 School of Medicine, Clinical Science Institute, National University of Ireland, Galway, Ireland; 2 School of Mathematics, Statistics and Applied Mathematics, National University of Ireland, Galway, Ireland; 3 HRB Clinical Research Facility, National University of Ireland, Galway, Ireland; Health Canada and University of Ottawa, Canada

## Abstract

**Background:**

Dicer, an RNase III-type endonuclease, is the key enzyme involved in RNA interference and microRNA pathways. Aberrant expression of Dicer is reported in several human cancers. Our aim was to assess the prognostic role of Dicer in breast cancer.

**Methods:**

The entire series comprised 666 invasive breast cancers (IBCs), 480 DCIS cases (397 associated with IBC and 83 pure DCIS) and 305 lymph node metastases. Cytoplasmic Dicer expression by immunohistochemistry was scored as negative (no staining) and positive (weak, moderate or strong staining).

**Results:**

Dicer staining was assessable in 446 IBC, 128 DCIS and 101 lymph node metastases. Expression of Dicer was observed in 33% (145/446) of IBCs, 34% (44/128) of DCIS and 57% (58/101) of lymph node metastases. Dicer expression was increased in nodal metastases compared to primary tumours (p<0.001); and was associated with ER negativity (p<0.001), HER2 positivity (p<0.001), high Ki67 labeling index (p<0.001) and expression of basal-like biomarkers (p = 0.002). Dicer positivity was more frequent in the HER2 overexpressing (p<0.001) and basal-like (p = 0.002) subtypes compared to luminal A subtype. Dicer expression was associated with reduced overall survival (OS) on univariate analysis (p = 0.058) and remained an independent predictor of OS on multivariate analysis (HR 2.84, 95% CI 1.43–5.62, p = 0.003), with nodal status (HR 2.61, 95% CI 1.18–5.80, p = 0.018) and PR (HR 0.28, 95% CI 0.13–0.59, p = 0.001). Further, moderate or strong expression of Dicer was associated with improved disease-free survival in the HER2-overexpressing subtype compared to negative or weak expression (p = 0.038).

**Conclusion:**

Deregulated Dicer expression is associated with aggressive tumour characteristics and is an independent prognostic factor for OS. Our findings suggest that Dicer is an important prognostic marker in breast cancer and that its prognostic role may be subtype specific.

## Introduction

MicroRNAs (miRNAs) are a family of short (∼20–23 nucleotide), endogenous, single-stranded RNA molecules that regulate gene expression in a sequence-specific manner [1]. Drosha and Dicer are two key enzymes involved in biogenesis of miRNAs. Following transcription, the primary miRNA transcript (pri-miRNA) undergoes endonucleocytic cleavage by the Drosha into a ∼60–100 nt hairpin precursor-miRNA (pre-miRNA) [2]. Following cleavage by Drosha, the pre-miRNA is transported out of the nucleus through interaction with exportin-5 (XPO5) and Ran-GTP [3] via recognition of the ∼2 nt 3′ overhangs of pre-miRNA hairpins [3]. In the cytoplasm, pre-miRNA is cleaved by RNase III enzyme Dicer, into a ∼22 nt double-stranded RNA product containing the mature miRNA guide strand and the passenger (miRNA*) strand. Mature miRNAs and Argonaute (Ago) proteins constitute the RNA-induced silencing complex (RISC), a ribonucleo-protein complex mediating post-transcriptional gene silencing [4]. Deregulation of miRNAs is associated with a broad range of human diseases including cancers [5] and miRNAs have been shown to be critically involved in control of cell survival and cell death decisions [6]. Global down-regulation of microRNAs (miRNAs) is a common feature of human tumours and impairment of miRNA biogenesis has been shown to enhance cancer progression [7].

The several components of miRNA biogenesis machinery have been shown to act as haploinsufficient tumour suppressors. For example, inactivating mutations have been reported in exportin-5 (*XPO5*) and TAR RNA-binding protein (*TRBP2*) in sporadic and hereditary colon carcinomas with microsatellite instability [8], [9]. Germline-inactivating mutations in Dicer have been shown to contribute significantly to familial pleuropulmonaryblastoma, cystic nephroma, ovarian Sertoli-Leydig tumour and intraocular medulloepithelioma [10], [11]. Analysis of eight tumours from Dicer mutation-positive patients showed there was no loss of the wild-type allele in any tumour [10]. These data suggest that Dicer down-regulation rather than its complete loss-of-function is selected for during tumourigenesis. Indeed, Dicer has been shown to act as a haploinsufficient tumour suppressor in K-Ras-induced mouse model of lung cancer and pre-clinical mouse model of retinoblastoma [12], [13]. Furthermore, analysis of Dicer copy number using data from Cancer Genome Project at the Sanger Institute revealed hemizygous deletions of *DICER1* in 27% (207/761) of tumours derived from tissues of diverse origins such as central nervous system, lung, pancreas, soft tissues, breast and bone [12] and hemizygous deletion of Dicer was also observed in approximately 37% of breast cancers [12]. Consistent with Dicer being a haploinsufficient tumour suppressor, homozygous deletions have not been observed in any of these 761 tumours [10].

Several studies have investigated the role Dicer in cancer tissues from different sites and aberrant expression is commonly reported. However in breast cancer, the role of Dicer in progression and behaviour is unclear. Dicer mRNA has been more extensively studied than protein in invasive breast cancer (IBC) [14]–[20] and some report an association between reduced mRNA levels and poor outcome [15] whereas others do not [16], [18]. Reports of the prognostic role of Dicer protein are similarly contradictory with some demonstrating an association between reduced expression and outcome [17] and others failing to show an association [15]. The aim of this study is to investigate Dicer protein expression in breast cancer and to explore its association with progression of disease, clinico-pathological features and outcome in a large series of IBC. We demonstrate herein that deregulated Dicer expression is significantly associated with several adverse clinical features such as ER negativity, Ki67 labelling index and expression of basal markers. We report that deregulated Dicer expression is associated with poor overall survival in IBC and is associated with a reduced disease free survival in the HER2 overexpressing subtype of breast cancer.

## Materials and Methods

### Ethics Statement

Use of patient material in this study was approved by the Clinical Research Ethics Committee, Merlin Park Hospital, Galway at the meeting of 18th January 2006 (reference CA 41). The ethics committee waived the need for consent.

### Patients and Tumour Material

Tumour material from 749 consecutive breast cancer cases managed at Galway University Hospital from 1999 to 2005 wasused for this study. Tissue microarrays (TMAs) were constructed from a single core (0.6 mm diameter) of formalin-fixed paraffin-embedded tissue as described previously [21]. The series comprised 666 invasive breast cancers, 480 DCIS cases (397 associated with IBC and 83 pure DCIS) and 305 lymph node metastases. The presence of tumour was confirmed by haematoxylin and eosin staining of TMA sections. The clinico-pathological characteristics of the series are shown in Table 1. Pathological data was taken from the original pathology report and all cases were reported by specialist breast pathologists. Grading of IBC was performed according to Elston and Ellis’ modified Bloom Richardson system [22] and DCIS was graded based on nuclear pleomorphism [23]. One hundred and thirteen patients diagnosed with IBC died during follow-up. Of the surviving patients, 301 had no evidence of disease progression. The median follow-up time was 48 months (range 1 to 177 months).

**Table 1:** Clinico-pathological features and biomarker profile of the series.

Parameter	n^a^		n^a^ (%)
**IBC**			
**Tumour grade**	**601**		
		1	82 (14)
		2	307 (51)
		3	212 (35)
**Tubule formation score**	**583**		
		1	31 (5)
		2	74 (13)
		3	478 (82)
**Nuclear pleomorphism score**	**583**		
		1	7 (1)
		2	287 (49)
		3	289 (50)
**Mitotic count score**	**583**		
		1	367 (3)
		2	103 (18)
		3	113 (19)
**Histological type**	**661**		
		Ductal	509 (77)
		Lobular	102 (15)
		Mucinous	14 (2)
		Mixed ductal and lobular	10 (2)
		Tubular	5 (1)
		Medullary	5 (1)
		Microinvasive ductal	5 (1)
		Apocrine	3 (0.5)
		Ductal and papillary or cribriform	3(0.5)
		Invasive papillary	2 (0.3)
		Other	3 (0.5)
**Tumour size (mm)**	**625**		
		≤20	239 (38)
		21–50	314 (50)
		>50	72 (12)
**Nodal status**	**596**		
		Positive	302 (51)
		Negative	294 (49)
**Number of positive lymph nodes**	**596**		
		0	294 (49)
		1–3	165 (28)
		4–9	88 (15)
		≥10	49 (8)
**Lymphovascular invasion**	**386**		
		Present	203 (53)
		Absent	174 (45)
		Probable	9 (2)
**Number of events**	**663**		
		Alive, no disease	301 (45)
		Alive, LR disease	191 (29)
		Alive, distant metastases	58 (9)
		Dead with disease progression	87 (13)
		Dead with no disease progression	26 (4)
**ER**	**522**		
		Positive	344 (66)
		Negative	178 (34)
**PR**	**534**		
		Positive	297 (56)
		Negative	237 (44)
**HER2**	**602**		
		Positive	85 (14)
		Negative	517 (86)
**BCL2**	**534**		
		Positive	290 (54)
		Negative	244 (46)
**Ki67**	**540**		
		Positive	163 (30)
		Negative	377 (70)
**EGFR**	**527**		
		Positive	77 (14)
		Negative	450 (85)
**CK5/6**	**463**		
		Positive	42 (9)
		Negative	421 (91)
**CK14**	**444**		
		Positive	94 (21)
		Negative	350 (79)
**p53**	**523**		
		Positive	105 (20)
		Negative	418 (80)
**DCIS associated with IBC**	**397**		
**Grade**	**339**		
		Low	13 (4)
		Intermediate	137 (40)
		High	189 (56)
**Size** b **(mm)**	**59**		
		Median	38
		Range	2–236
**Pure DCIS**	**83**		
**Grade**	**54**		
		Low	7 (13)
		Intermediate	21 (39)
		High	26 (48)
**Size (mm)**	**41**		
		Median	15
		Range	0.8–83
**Number of events**	**81**		
		Alive, no disease	63 (78)
		Alive, LR disease	4 (5)
		Alive, distant metastases	1 (1)
		Dead with disease progression	9 (11)
		Dead with no disease progression	4 (5)

^a^ no. of cases for which data is available.

^b^ whole tumour measurement is given in cases if it differs from the size of the IBC.

LR: locoregional.

### Immunohistochemistry

TMA sections of IBC were stained with oestrogen receptor (ER) (Neomarker, clone SP, 1∶100), progesterone receptor (PR) (Leica, clone 16, 1∶200), HER2 (Dako), Ki67 (Dako, MIB-1, 1∶200), BCL2 (Novocastra, bcl-2/100/D5, 1∶200), cytokeratin (CK) 14 (Novocastra, LL002, 1∶20), CK5/6 (Dako, D5/16, 1∶100), EGFR (Dako, pharmDx kit) and p53 (Dako, DO-7, 1∶100) using standard indirect immunoperoxidase procedures. Nuclear staining for ER, PR [24]–[27], p53 [28]–[30] and Ki67 was scored as positive with a cut-off of 10% [31]–[36]. Membranous staining with HER2 was recorded as negative, weakly positive and positive (Dako Herceptest method) [37] with confirmation of 2+ cases by fluorescent in situ hybridisation (FISH). Cytoplasmic expression of BCL2 with a 10% cut-off was used [38]–[40] and any observed cytoplasmic staining with CK14, CK5/6 and EGFR was considered positive. Molecular subtypes were defined based on these markers as luminal A (ER and/or PR positive, HER2 negative); luminal B (ER and/or PR positive, HER2 positive); HER2-overexpressing (ER and PR negative, HER2 positive); triple negative (ER, PR and HER2 negative); and basal-like (ER, PR, HER2 negative and positive for at least one basal marker (CK5/6, CK14 or EGFR)). IHC was scored by two pathologists independently each blinded to the clinico-pathological data and outcome (Table 1).

**Figure 1:**
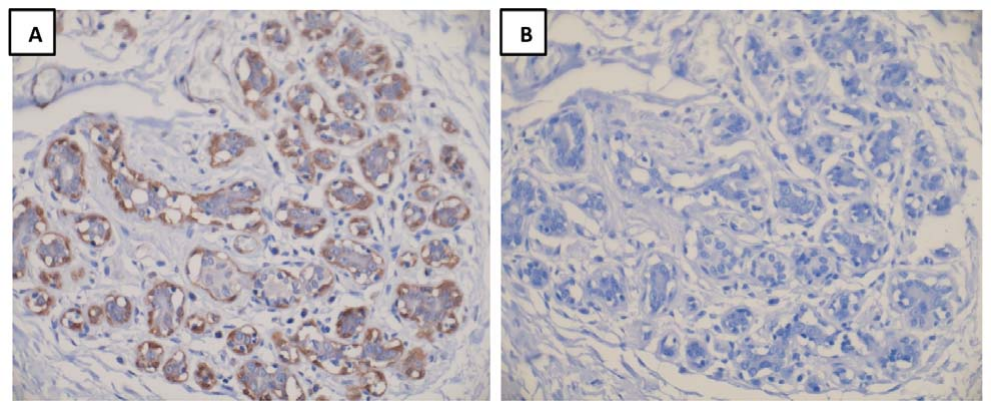
Dicer expression by IHC in normal breast tissue. Representative images of Dicer staining (A) without blocking peptide and (B) with blocking peptide are shown. Panel A is representative of Dicer staining in normal breast tissue.

We tested two commercially available anti-Dicer antibodies [Clonegene rabbit polyclonal antibody, (CG031, clone 13D6R) and Abcam mouse monoclonal antibody, (ab14601, clone 13D6)]. We observed specific myoepithelial staining with the Clonegene antibody (Figure 1a) as reported previously [19]. We confirmed the specificity of the Clonegene antibody by pre-incubation with an excess of its competing peptide (CG302, Clonegene) and staining of breast whole tissue sections (WTS) (Figure 1b). A blocking peptide against the Abcam anti-Dicer antibody was not available. The anti-Dicer antibody from Clonegene (CG301) was used for subsequent analysis. TMA sections were cut at 4 µm thickness and mounted onto glass slides, deparaffinised in xylene, followed by hydration in graded ethanol solutions. Antigen retrieval involved microwaving of sections in citrate buffer (pH 6) at 98°C for 20 mins. Sections were blocked for 10 mins in 2% horse serum in 0.1 M phosphate buffered saline. Slides were then incubated with anti-Dicer antibody at a dilution of 1∶2500 at room temperature overnight. After washing, ImmPRESS universal reagent (Vector) was applied for 40 minutes. The chromogen was then developed with ImmPACT DAB (Vector). A haematoxylin counterstain was used.

**Figure 2:**
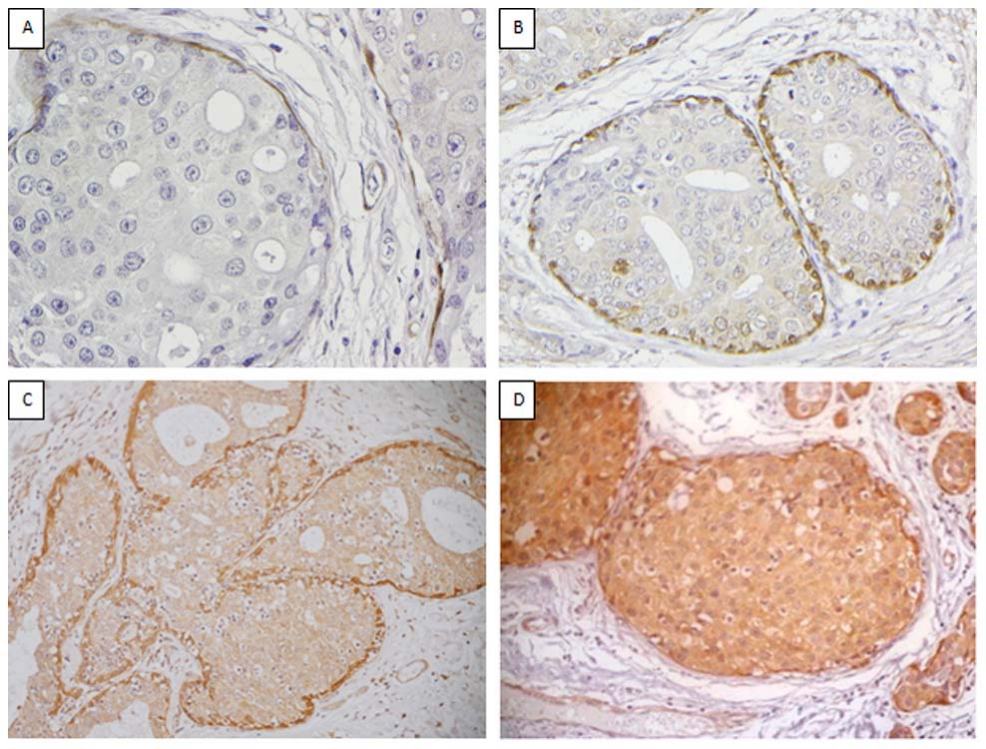
Dicer expression in DCIS. Representative images of the spectrum of the staining intensity observed for Dicer in DCIS where 0 = negative (A), 1 = weak (B), 2 = moderate (C) and 3 = strong (D).

**Figure 3:**
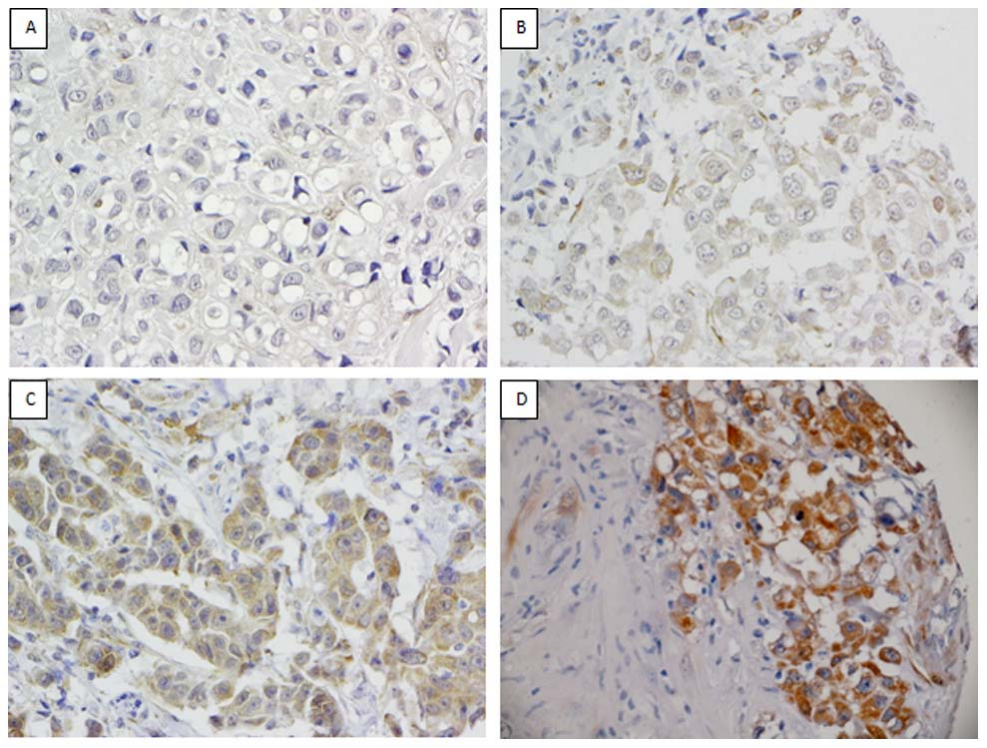
Dicer expression in IBC. Representative images of the spectrum of staining observed for Dicer in IBC where 0 = negative (A), 1 = weak (B), 2 = moderate (C) and 3 = strong (D).

Scoring of Dicer was optimised on 26 WTSs of IBC. Dicer was expressed consistently in the cytoplasm of myoepithelial cells (Figure 1b). Luminal epithelium was negative. Cases of IBC, pure DCIS and DCIS associated with IBC and lymph node metastases were stained with Dicer. Tumour cells showed cytoplasmic expression with nuclear staining rarely observed in cases with moderate or strong cytoplasmic expression. Cores of normal breast parenchyma and normal tissue within tumour cores served as positive controls. Intensity of cytoplasmic staining was scored as 0, absent; 1, weak; 2, moderate; or 3, strong staining (Figures 2 and 3), where score 0 and score 3 showed expression equal to that seen in benign luminal cells and in normal myoepithelial cells respectively. The percentage of positive tumour cells was recorded and was homogeneous throughout each tumour.

### Statistical Analysis

Since the staining for Dicer was homogeneous, only intensity of Dicer staining was used for analysis as reported by others [41]–[45]. Cases with no staining (score 0) were considered negative and cases with any staining intensity (scores 1 to 3) were considered positive. This cut off was used because the number of cases with an intensity score of 2 and 3 were quite small and it also demonstrated the strongest association with outcome. Cases without a representative stained core were excluded from the analysis. Summary statistics included proportions for categorical variables. Comparisons between Dicer expression and clinico-pathological features were initially analysed using Chi-square tests. p values <0.0025 were considered significant when the Bonferroni correction for multiple tests was applied. Two proportion tests were then used to estimate the effect (difference in proportions) of significant variables. Kaplan-Meier estimates were plotted for overall survival (OS) and disease-free survival (DFS). The log-rank test was used to examine the statistical significance of the differences observed between the groups. A multivariate Cox regression model was also used to compute hazard ratios (HR) and 95% confidence intervals (95% CI), adjusting for known prognostic variables (including grade, tumour size, nodal status). Stepwise variable selection was used to identify the most parsimonious model with Dicer expression which best predicted DFS and OS. p values reported were two tailed and p<0.05 was considered statistically significant. Statistical analysis was performed using R statistical software (v2.12.0) and SPSS (v20).

## Results

### Dicer Expression in Breast Cancer Progression

Data on Dicer expression by immunohistochemistry was available in 446 IBCs, the associated DCIS in 108 cases, 20 cases of pure DCIS and 101 lymph node metastases. The number of pure DCIS cases was small, therefore for analysis these cases were combined with data on DCIS with associated IBC (total = 128). Expression of Dicer (scores 1, 2 or 3) was observed less frequently in DCIS (44/128, 34%), and IBC (145/446, 33%) compared with lymph node metastasis (58/101, 57%) (χ^2^ = 22.37, p<0.001) (Table 2). There was no association between Dicer staining and grade of DCIS and there was no difference in Dicer expression comparing pure DCIS to DCIS with associated IBC. Dicer was expressed in 36% (n = 24) of high grade, 23% (n = 11) of intermediate grade and 50% (n = 1) of low grade DCIS (p = 0.23, Fisher’s exact test). Dicer expression was examined in paired samples. There was no difference between expression of Dicer in DCIS and IBC in 42 of 65 (14 positive and 28 negative) of paired cases. However, up-regulation of expression between DCIS (score 0) and invasive tumour (score 1, 2 or 3) was seen in 20% (n = 13) while 15% (n = 10) showed the converse. Next we analysed 76 paired IBCs and lymph node metastases. Dicer expression was unchanged in 42 (55%) paired cases (15 positive and 27 negative), and was negative in IBC and positive in lymph node metastasis in 26 (34%) cases while 8 (11%) showed the converse.

**Table 2:** Dicer expression scores in invasive carcinoma, DCIS and lymph node metastasis.

Dicer Score (intensity)	DCIS	Invasive Carcinoma	Lymph Node Metastasis
	n^a^(%)	n^a^(%)	n^a^(%)
Negative (0)	84 (66)	301 (67)	43 (43)
Positive (1,2,3)	44 (34)	145 (33)	58 (57)
Intensity			
1	28 (22)	76 (17)	42 (42)
2	15 (12)	40 (9)	12 (12)
3	1 (1)	29 (7)	4 (4)
Total	128^b^	446	101

^a^ number of cases for which Dicer expression data was available.

^b^ includes 108 DCIS cases associated with DCIS and 20 pure DCIS cases.

### Dicer Expression in IBC and its Associations with Clinico-pathological Features

Associations between Dicer expression and clinico-pathological variables are shown in Table 3. Dicer positivity (score 1, 2 or 3) was seen in a significantly higher proportion of ER negative as compared to ER positive cases (estimate of difference in proportions of 27.4%). There was a significant association between Dicer positivity and HER2 overexpression (estimate of difference in proportions of 20.3%) and Ki67 labelling index. Dicer positivity was also associated with expression of basal-like biomarker EGFR and, in the analysis of breast cancer subtypes, luminal A subtype showed a significantly lower proportion of Dicer positive patients than basal-like and HER2 overexpressing subtypes.

**Table 3:** Associations between Dicer expression and clinico-pathological variables in IBC.

Variable	Total	Dicer positive (Intensity 1,2,3)	Dicer negative (Intensity 0)	?^2^	p value^b^
	n^a^ (%)	n^a^ (%)	n^a^ (%)		
**Patient age (years)**					
<50	131 (29)	44 (34)	87 (66)	0.10	0.754
≥50	315 (71)	101 (32)	214 (68)		
**Histological tumour type**					
Ductal	345 (78)	119 (34)	226 (66)	2.58	0.275
Lobular	72 (16)	18 (25)	54 (75)		
Other	24 (6)	7 (29)	17 (71)		
**Tumour grade**					
1/2	305 (69)	88 (29)	217 (71)	9.07	0.024
3	134 (31)	56 (42)	78 (58)		
**Tubule formation score**					
1	25 (6)	6 (24)	19 (76)	1.75	0.417
2	52 (13)	20 (38)	32 (62)		
3	335 (81)	106 (32)	229 (68)		
**Nuclear pleomorphism score**					
1	5 (1)	1 (20)	4 (33)	2.52	0.284
2	208 (51)	60 (29)	148 (56)		
3	199 (48)	71 (36)	128 (64)		
**Mitotic count score**					
1	265 (64)	74 (28)	191 (72)	8.66	0.007
2/3	147 (36)	58 (39)	89 (61)		
**UICC** c **T stage**					
pT1	121 (28)	30 (25)	91 (75)	4.52	0.104
pT2	249 (58)	89 (36)	160 (64)		
pT3	62 (14)	21 (34)	41 (66)		
**UICC** c **N stage**					
pN0	210 (49)	71 (34)	139 (66)	1.47	0.681
pN1	119 (28)	36 (30)	83 (70)		
pN2	65 (15)	23 (35)	42 (65)		
pN3	32 (8)	8 (25)	24 (75)		
**Lymphovascular invasion**					
Present	144 (54)	46 (32)	98 (68)	0.59	0.444
Absent	123 (46)	34 (28)	89 (72)		
**ER status**					
Positive	274 (67)	66 (24)	208 (76)	30.31	<0.001
Negative	132 (33)	68 (52)	64 (48)		
**PR status**					
Positive	236 (57)	69 (29)	167 (71)	3.37	0.066
Negative	180 (43)	68 (38)	112 (62)		
**HER2**					
Positive	64 (14)	32 (50)	32 (50)	10.23	0.001
Negative	380 (86)	113 (30)	267 (70)		
**Triple negative**					
Yes	57 (14)	28 (49)	29 (51)	8.47	0.004
No	355 (86)	106 (30)	249 (70)		
**Molecular subtype**					
Luminal A	277 (76)	71 (26)	206 (74)		
Luminal B	27 (7)	10 (37)	17 (63)	1.11	0.293^d^
HER2 overexpressing	34 (9)	21 (62)	13 (38)	17.29	<0.001^d^
Basal-like	27 (7)	15 (56)	12 (44)	9.43	0.002^d^
**Ki67 labelling index**					
<10%	294 (71)	79 (27)	215 (73)	12.16	<0.001
≥10%	119 (29)	53 (45)	66 (55)		
**BCL2**					
Positive	226 (55)	64 (28)	162 (72)	3.18	0.074
Negative	186 (45)	68 (37)	118 (63)		
**CK14**					
Positive	80 (21)	34 (43)	46 (57)	5.04	0.025
Negative	297(79)	87 (29)	210 (71)		
**CK5/6**					
Positive	52 (14)	21 (40)	31 (60)	1.15	0.285
Negative	326 (86)	107 (33)	219 (67)		
**EGFR**					
Positive	54 (13)	31 (57)	23 (43)	17.69	<0.001
Negative	356 (87)	102 (29)	254 (71)		
**p53**					
Positive	83 (21)	37 (45)	46 (55)	6.75	0.009
Negative	318 (79)	94 (30)	224 (70)		

^a^ number of cases for which Dicer expression and data for the relevant parameter was available.

^b^ p values <0.0025 were considered significant when the Bonferroni correction for multiple tests was applied.

^c^ UICC TNM Classification of Malignant Tumours 7^th^ Edition [70].

^d^ compared to luminal A subtype.

### Dicer Expression and Patient Outcome

**Figure 4:**
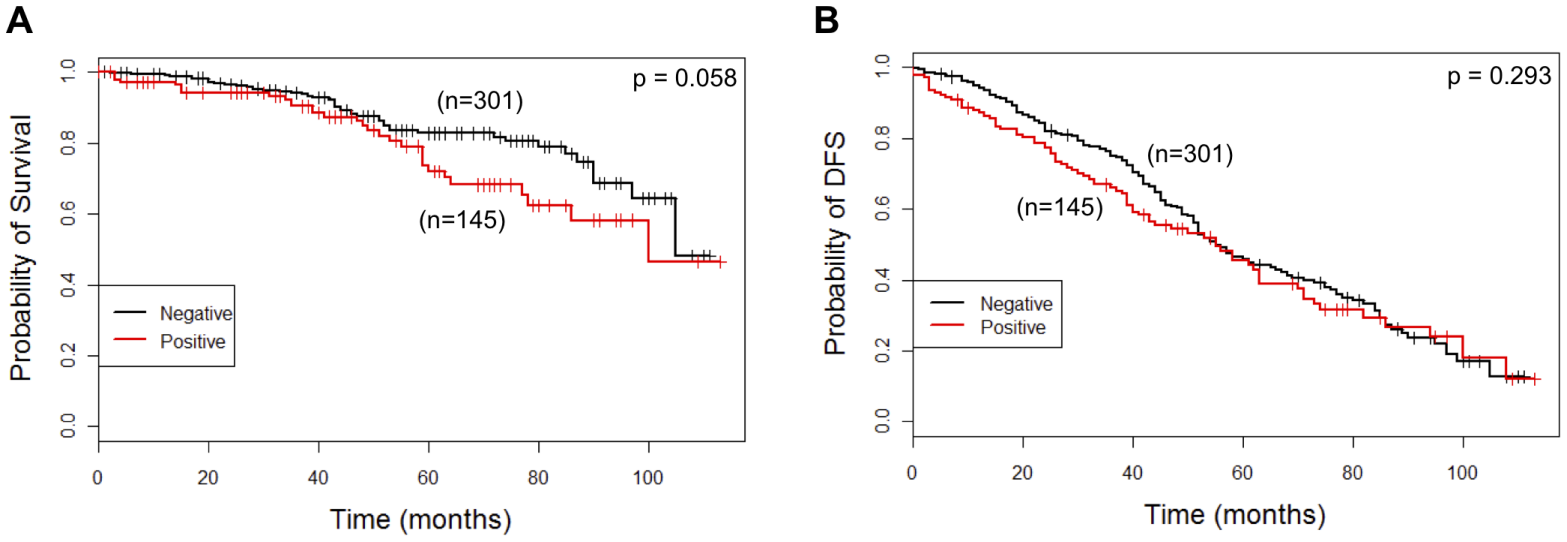
Kaplan-Meier curves for OS (A) and DFS (B) in IBC categorised according to Dicer expression in the entire series. Dicer expression is categorised as negative (intensity score 0) and positive (intensity score 1, 2 or 3).

In the whole series, Dicer expression (score 1, 2 or 3) was associated with reduced overall survival (OS) compared with Dicer negativity by Kaplan Meier estimates (p = 0.058, log-rank test) (Figure 4a) but there was no significant difference in disease free survival (DFS) between those who were Dicer positive or negative (Figure 4b). Cox regression revealed that Dicer positivity was associated with an increased likelihood of death of 1.55 (CI: 0.98–2.45) compared with Dicer negativity (p = 0.061). Multivariate Cox proportional hazard regression analysis, adjusting for lymph node status, pathological T stage, tumour grade, lymphovascular invasion, ER, PR and HER2 status, revealed that Dicer expression was a strong (significant) predictor for OS (p = 0.003). In the final model, selected using variable selection, Dicer expression was associated with an increased likelihood of death from breast cancer by a factor of 2.84 (HR, 2.84; 95% CI, 1.43–5.62; p = 0.003) adjusting for lymph node status and PR status (Table 4). Dicer expression in IBC was not a significant predictor of DFS in the multivariate analysis, neither was Dicer expression in the lymph node metastases associated with OS or DFS (p = 0.57 and p = 0.15 respectively, log-rank test).

**Table 4:** Multivariate analysis of Dicer expression for OS.

Variable	Coefficient	HR^a^	95% CI	p value
Dicer expression	1.04	2.84	1.43–5.62	0.003
Lymph node status	0.96	2.61	1.18–5.80	0.018
PR status	−1.28	0.28	0.13–0.59	0.001

^a^ HR: Hazard ratio.

**Figure 5:**
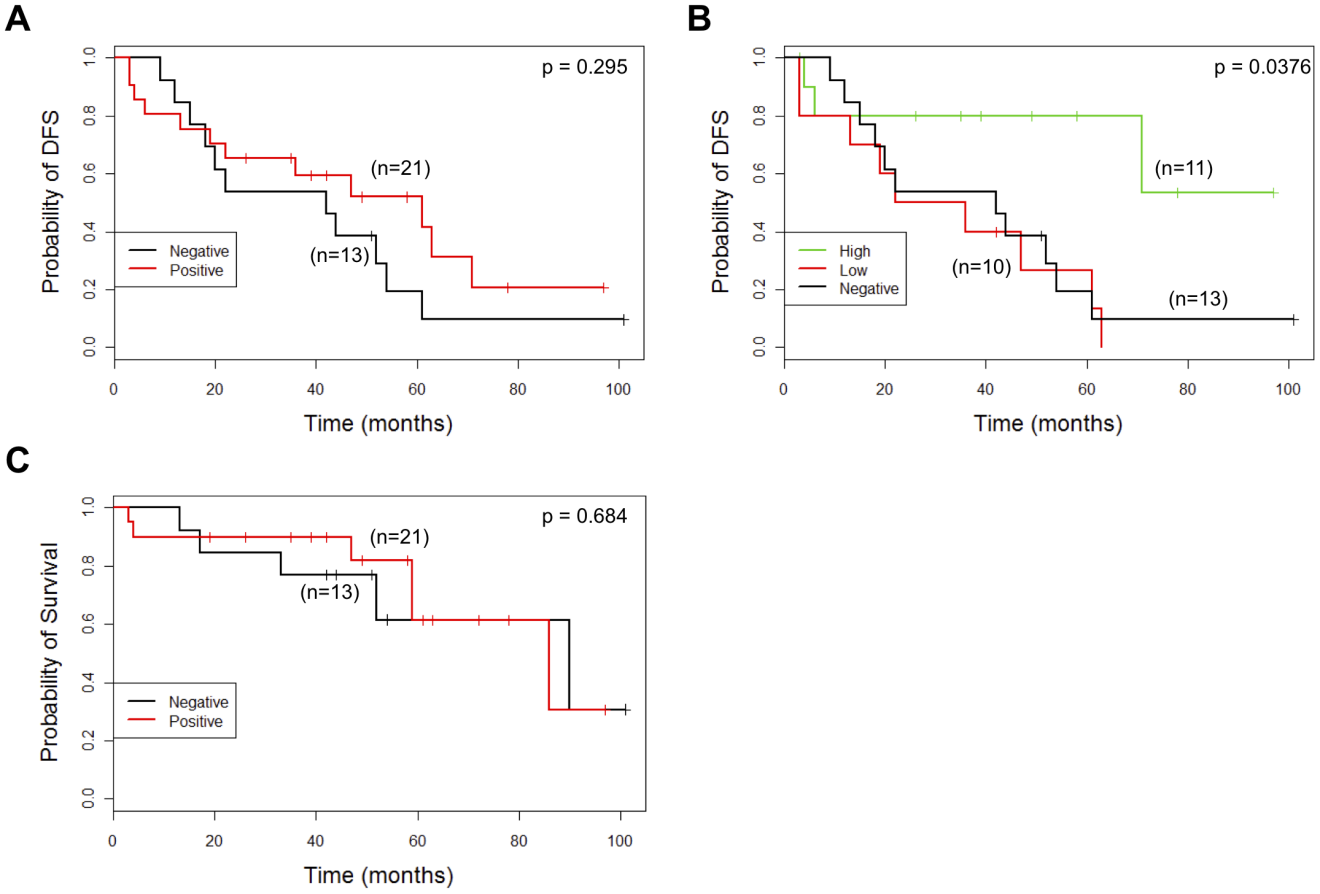
Kaplan-Meier curves for DFS (A, B) and OS (C) in the HER2 overexpressing subtype of IBC categorized according to Dicer expression. (A, C) Dicer expression is categorised as negative (intensity score 0) and positive (intensity scores 1, 2 and 3). (B) Dicer expression is categorised as negative (intensity score 0), low (intensity score 1) and high (intensity score 2 and 3).

Finally, we examined the association between Dicer expression and outcome in the different molecular subtypes as Dicer expression was significantly associated with HER2 overexpressing and basal-like subtypes on univariate analysis (Table 3). Dicer expression was associated with a poorer OS compared with Dicer negativity in the luminal B subtype (p = 0.057, log-rank test) but was not significant for the other subtypes (Figure A in file Figure S1). Neither was there a significant association between Dicer expression and DFS in the different subtypes (Figure A in file Figure S1). Because Dicer expression appeared to be paradoxically associated with improved DFS in the HER2 overexpressing subtype (shown in Figure 5a), we examined this association further. For this purpose the Dicer-positive group was divided into Dicer low (score 1) and Dicer high (score 2 or 3) categories. Although the number of cases and events at extended follow up are small, we observed a better prognosis for DFS with high Dicer expression compared to low or negative Dicer expression in the HER2 overexpressing subgroup (p = 0.0376) (Figure 5b).

### Association between High Dicer Expression and Clinico-pathological Variables and Outcome

Associations between high Dicer expression and clinico-pathological variables, biomarker expression and outcome were also examined by dichotomising Dicer expression as low (intensity scores 0 and 1) and high (intensity scores 2 and 3). Again, there was a significant association between high Dicer expression and the HER2 overexpressing compared to the luminal A subtype (χ^2^ = 8.31, p = 0.004); and high Dicer expression was more common in HER2 positive (χ^2^ = 4.29, p = 0.04) and EGFR positive (χ^2^ = 4.73, p = 0.03) compared to HER2 and EGFR negative cases respectively. No other statistically significant associations between high Dicer expression and clinico-pathological variables, biomarker expression, or outcome were found, perhaps due to the lower numbers of cases in the score 2/3 group (n = 69) (Supplementary Table S1).

## Discussion

We examined expression of the Dicer protein, a critical enzyme involved in miRNA biogenesis, in breast cancer with the aim of exploring its role in disease progression and its impact on outcome. We found that Dicer expression was similar in the majority of in situ and invasive tumours and was increased in nodal metastases suggesting a role for in Dicer in progression to metastatic disease. Dicer expression was associated with features of aggressive disease and was a predictor of reduced OS that was independent of clinico-pathological variables, steroid hormone and HER2 receptor status in the entire series. Paradoxically, low Dicer expression was significantly associated with reduced DFS in HER2 expressing subtype of disease.

Deregulation of Dicer is commonly observed in several tumour types however there are conflicting results regarding its prognostic role. Increased Dicer protein expression was associated with adverse prognostic features in cancers of the prostate [41], ovary [42], colon [44], in malignant melanomas [45], soft tissue sarcomas [46] and nasopharyngeal carcinomas [47], whereas low protein expression was associated with tumour invasiveness, metastasis and poor survival in carcinomas of the gallbladder [48], colon [49], ovary [50], and breast [17]. Results from studies of Dicer mRNA across tumour types are also contradictory. Reduced Dicer mRNA in cancers of the breast [14]–[16], [51]–[53], lung [54], [55] and ovary [56] was associated with aggressive phenotypic features, whereas the converse is described in prostatic [41], ovarian [42], [57], oesophageal [58] and colorectal [44] cancers by others. The data for Dicer is also conflicting within tumours of the same type. We show a paradoxical association for Dicer protein and outcome in the HER2 overexpressing subtype of breast cancer compared to the whole series. In ovarian cancer, Flavin et al. found an association between increased Dicer protein expression and both nodal metastasis and high Ki67 proliferation index [42], while Faggad et al. observed low levels of Dicer in tumours with lymph node metastasis, high grade and poor survival [50]. Similarly, both low [49] and high [44] Dicer protein expression were associated with and adverse features and/or outcome in colorectal cancer.

In IBC, Dicer mRNA has been evaluated more commonly than protein and an association between low mRNA levels and adverse clinico-pathological features and/or adverse outcome is reported by most [14]–[16], [51], [52], [59] but not all [20]. Only two other reports have evaluated the prognostic role of Dicer protein in breast cancer tissue samples [15], [17]. Grelier et al. found that low mRNA levels were associated with non-luminal subtype and reduced metastases free survival whereas low protein expression was significantly associated with luminal A subtype and not with outcome in 86 cases [15], [17]. The present study is one of only two reports that examined Dicer protein in a large patient series and both show that Dicer is an independent predictor of outcome. In a series of over 1000 IBCs, Khoshnaw et al. showed that reduced protein expression was an independent predictor of improved DFS and was significantly associated with high tumour grade, hormone receptor negativity and absence of luminal keratins [17]. The pattern of Dicer expression in that study however conflicts both with data from our study, where Dicer positivity was associated with aggressive phenotypic features (high Ki67, ER negativity, basal phenotype and with reduced OS) and also with the data from Grelier et al. where Dicer protein was associated with ER positivity and the luminal A subtype [15], [17].

Several reasons have been proposed for these discrepancies. miRNA expression patterns are highly specific for cell type and cellular differentiation status. Thus, depending on whether the net effect of the majority of miRNAs in a given cell is oncogenic or tumour suppressive, the loss of Dicer expression can have opposite consequences on cell survival and proliferation. Thus, Dicer deregulation may be site specific and its role may differ in different tumours and in different subtypes. This is supported by functional studies where knockdown of Dicer expression rendered MDAMB-231 and MDA-MB-436 cells significantly more invasive, while knockdown of Dicer in MCF-7 cells led to G1 arrest and increase sensitivity to cisplatin [60], [61] suggesting that the effects of Dicer on development and progression of cancer are context-dependent. Secondly, Dicer mRNA levels are not well correlated with protein levels with a 72% concordance reported in cell lines using microarray hybridisation and real-time reverse transcription-PCR (qPCR) [15]. Furthermore, the regulation of Dicer expression is largely post-transcriptional and complex. A regulatory feedback loop exists through which Dicer expression is regulated by mature miRNAs such as miR-103/107 and let-7 family members [18], [62].

Technical issues are also likely to be important. Unresolved issues about the specificity and sensitivity of commercially available anti-Dicer antibodies are a major limiting factor in the interpretation of protein expression by IHC. Different antibodies have been used by investigators and all describe cytoplasmic expression of Dicer. Notably, nuclear staining was seen in triple negative breast cancer in one report [19], and we also observed nuclear staining in a minority of samples where there was strong cytoplasmic staining. Nuclear expression would be consistent with a function of Dicer in the nucleus and, indeed, Dicer has been shown to shuttle between the cytoplasm and the nucleus in the fission yeast *Schizosaccharomyces pombe* and mammalian cells [63], [64]. In IBC both strong and weak staining was observed in normal luminal epithelial cells in two reports [15], [17] whereas our study and that of Passon et al. [19] observed strong staining in normal myoepithelial cells. Notably, the specificity of the antibody used by us was confirmed by pre-incubation with an excess of its competing peptide. Finally, the small size of series and different scoring systems employed by investigators compound the issue. Only six reports, including the present report, included more than 200 cases from cancers of the breast [17], stomach [43], colon [49], malignant melanomas [45] and nasopharyngeal carcinoma [47], and scoring systems based on the intensity of staining [15], [41]–[46], [54], [65]–[67] or the percentage of positive cells with [17], [47], [49], [50] or without [48] intensity have been employed. The extent to which these technical variations influence the interpretation of the clinical role of Dicer protein expression remains unclear.

We found that Dicer expression differed significantly between the primary tumour (either in situ or invasive) and the metastasis. A putative role for Dicer in neoplastic progression from normal to malignant to metastatic disease has been demonstrated in clinical studies at other sites including the prostate [41], ovary [57], lung [54] and colon [68] as well as breast [15], [17]. Khoshnaw et al. observed a gradual change in Dicer expression with progression from normal tissue, to either in situ or invasive disease to nodal metastasis [17] and Dicer mRNA levels differed between breast cancers with nodal metastases and those without [15]. While the precise level of Dicer expression differs between these studies, nonetheless they support a role for its deregulation in breast cancer progression. A putative mechanism through induction of a mesenchymal phenotype in breast cancer has been proposed. Dicer mRNA was lower in cell lines that underwent epithelial to mesenchymal transition (EMT) [15], [69] and down-regulation of Dicer protein by miR-103/107 was shown to be associated with EMT and metastasis [18].

In summary, we report the second largest study of Dicer protein expression in clinical breast cancer samples. We show that expression of Dicer protein is deregulated and stepwise alterations occur between in situ or invasive disease and nodal metastases. Deregulated, namely increased, expression was significantly associated with features of aggressive disease and was an predictor of reduced OS in independent of clinico-pathological variables, steroid hormone and HER2 receptor status the whole series whereas high Dicer expression was associated with an improved DFS in the HER2 overexpressing subgroup. There are clear discrepancies between reports about the precise expression level of Dicer mRNA and protein in clinical samples from tumours at the same and different sites and their correlation with clinico-pathological features and outcome. However, not withstanding the inconsistencies it is encouraging that Dicer was an independent predictor of outcome in the two largest series studied. These support a role for Dicer in progression of disease and in prognostication. Studies to further elucidate the complex mechanisms regulating expression of Dicer and to investigate its relationship with other factors and pathways in human cancer types are warranted. Furthermore, issues about the specificity and sensitivity of commercially available anti-Dicer antibodies need to be investigated further.

## Supporting Information

Figure S1 Figure A. Kaplan-Meier curves for OS in the different subtypes of IBC categorised according to Dicer expression. (A) Luminal A, (B) luminal B, (C) HER2 overexpressing, (D) triple negative and (E) basal-like. In each subtype, Dicer expression is categorised as negative (intensity score 0) and positive (intensity score 1, 2 and 3). Figure B. Kaplan-Meier curves for DFS in the different subtypes of IBC categorised according to Dicer expression. (A) Luminal A, (B) luminal B, (C) HER2 overexpressing, (D) triple negative and (E) basal-like subtype. In each subtype, Dicer expression is categorised as negative (intensity score 0) and positive (intensity score 1, 2 and 3).
(PPTX)

Table S1 Associations between Dicer expression dichotomised as positive (intensity score 2 and 3) and negative (intensity score 0, 1) and clinico-pathological variables in IBC.
(DOC)
