# Supplementary material for: Prognostic Significance of Deregulated Dicer Expression in Breast Cancer
Source: PLoS One. 2013 Dec 30;8(12):e83724. doi: 10.1371/journal.pone.0083724 (PMC3875475; doi:10.1371/journal.pone.0083724)
Supplement: Figure S1 — Figure A. Kaplan-Meier curves for OS in the different subtypes of IBC categorised according to Dicer expression. (A) Luminal A, (B) luminal B, (C) HER2 overexpressing, (D) triple negative and (E) basal-like. In each subtype, Dicer expression is categorised as negative (intensity score 0) and positive (intensity score 1, 2 and 3). Figure B. Kaplan-Meier curves for DFS in the different subtypes of IBC categorised according to Dicer expression. (A) Luminal A, (B) luminal B, (C) HER2 overexpressing, (D) triple negative and (E) basal-like subtype. In each subtype, Dicer expression is categorised as negative (intensity score 0) and positive (intensity score 1, 2 and 3). (PPTX) [file pone.0083724.s001.pptx]

## Slide 1
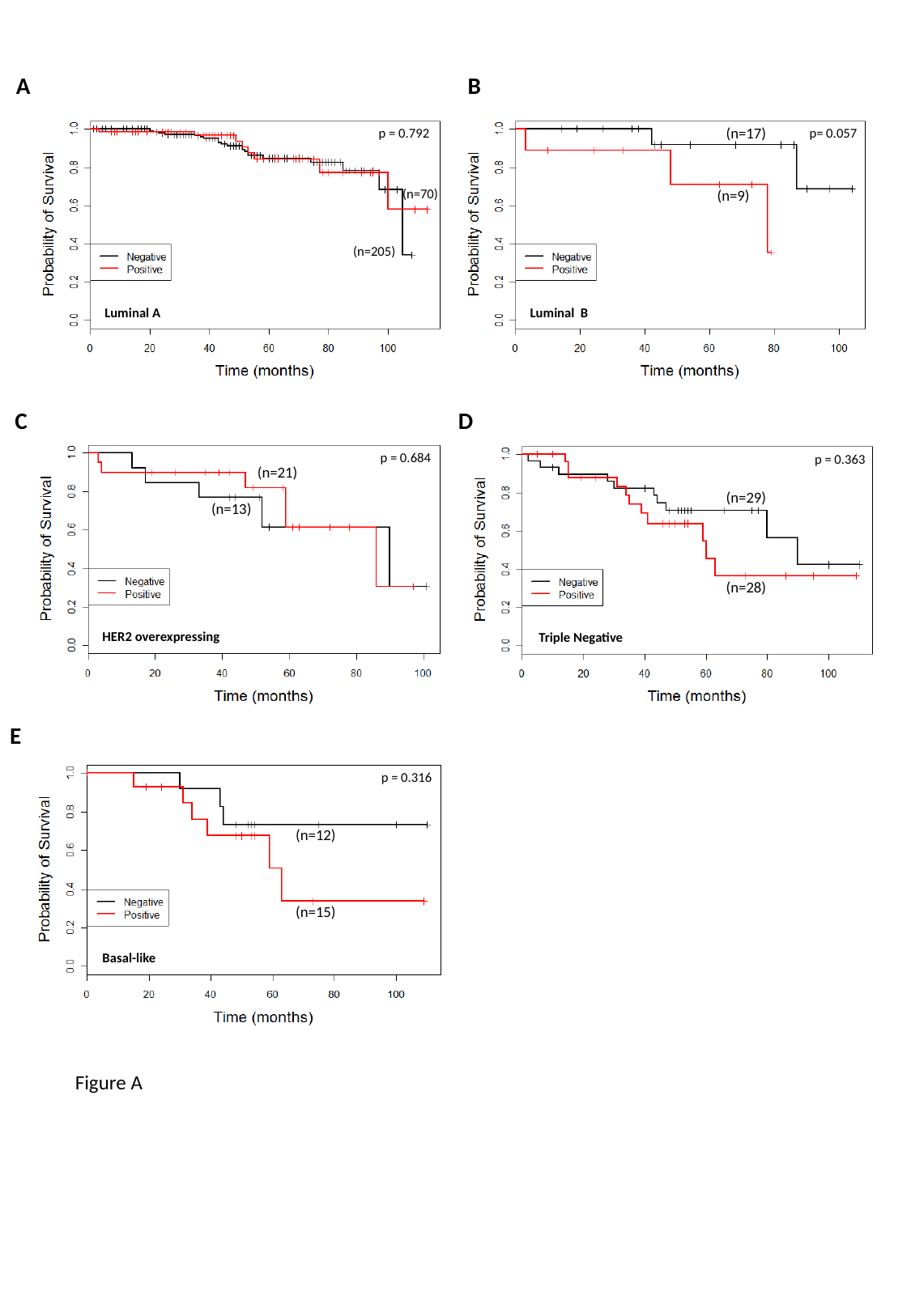

A
B
(n=17)
p = 0.792
p= 0.057
(n=70)
(n=9)
(n=205)
Luminal A
Luminal B
C
D
p = 0.684
p = 0.363
(n=21)
(n=29)
(n=13)
(n=28)
HER2 overexpressing
Triple Negative
E
p = 0.316
(n=12)
(n=15)
Basal-like
Figure A

## Slide 2
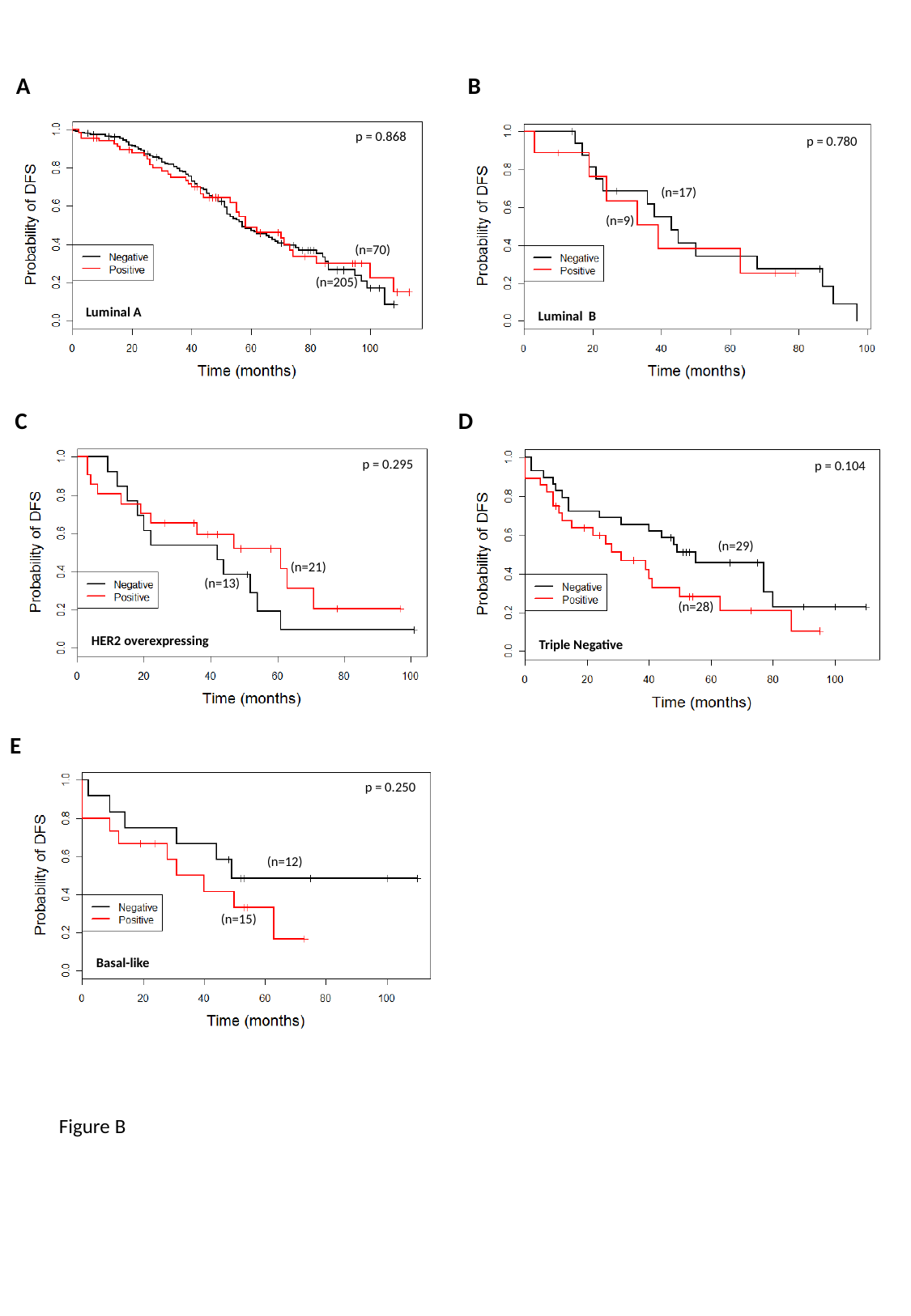

A
B
p = 0.868
p = 0.780
(n=17)
(n=9)
(n=70)
(n=205)
Luminal A
Luminal B
C
D
p = 0.295
p = 0.104
(n=29)
(n=21)
(n=13)
(n=28)
HER2 overexpressing
Triple Negative
E
p = 0.250
(n=12)
(n=15)
Basal-like
Figure B
