# Supplementary material for: Prognostic Significance of Deregulated Dicer Expression in Breast Cancer
Source: PLoS One. 2013 Dec 30;8(12):e83724. doi: 10.1371/journal.pone.0083724 (PMC3875475; doi:10.1371/journal.pone.0083724)
Supplement: Table S1 — Associations between Dicer expression dichotomised as positive (intensity score 2 and 3) and negative (intensity score 0, 1) and clinico-pathological variables in IBC. (DOC) [file pone.0083724.s002.doc]

**Table S1.** Associations between Dicer expression dichotomised as positive (intensity score 2 and 3) and negative (intensity score 0, 1) and clinico-pathological variables in IBC.

| **Variable** | **Total** | **Dicer positive**  **(Intensity 2, 3)** | **Dicer negative**  **(Intensity 0, 1)** | **χ^2^** | **p value^b^** |
| --- | --- | --- | --- | --- | --- |
|  | **n^a^(%)** | **n^a^(%)** | **n^a^ (%)** |  |  |
| **Patient age (years)** |  |  |  |  |  |
| **<50** | 131 (29) | 27 (21) | 104 (79) | 3.21 | 0.07 |
| **≥50** | 315 (71) | 42 (13) | 273 (87) |  |  |
| **Histological tumour type** |  |  |  |  |  |
| **Ductal** | 345 (78) | 58 (17) | 287 (83) | 2.46 | 0.29 |
| **Lobular** | 72 (16) | 7 (10) | 65 (90) |  |  |
| **Other** | 24 (6) | 3 (12.5) | 21 (87.5) |  |  |
| **Tumour grade** |  |  |  |  |  |
| **1/2** | 305 (69) | 43 (14) | 262 (86) | 1.15 | 0.28 |
| **3** | 134 (31) | 25 (19) | 109 (81) |  |  |
| **Tubule formation score** |  |  |  |  |  |
| **1** | 25 (6) | 5 (20) | 20 (80) | 3.24 | 0.20 |
| **2** | 52 (13) | 11 (21) | 41 (79) |  |  |
| **3** | 335 (81) | 43 (13) | 292 (87) |  |  |
| **Nuclear pleomorphism score** |  |  |  |  |  |
| **1** | 5 (1) | 0 (0) | 5 (100) | 1.22 | 0.54 |
| **2** | 208 (51) | 28 (13) | 180 (87) |  |  |
| **3** | 199 (48) | 31 (16) | 168 (84) |  |  |
| **Mitotic count score** |  |  |  |  |  |
| **1** | 265 (64) | 35 (13) | 230 (87) | 0.52 | 0.47 |
| **2/3** | 147 (36) | 24 (16) | 123 (84) |  |  |
| **UICC^c^ T stage** |  |  |  |  |  |
| **pT1** | 121 (28) | 20 (17) | 101 (83) | 0.11 | 0.95 |
| **pT2** | 249 (58) | 38 (15) | 211 (85) |  |  |
| **pT3** | 62 (14) | 10 (16) | 52 (84) |  |  |
| **UICC^c^ N stage** |  |  |  |  |  |
| **pN0** | 210 (49) | 34 (16) | 179 (84) | 0.55 | 0.91 |
| **pN1** | 119 (28) | 21(18) | 98 (82) |  |  |
| **pN2** | 65 (15) | 10 (15) | 55(85) |  |  |
| **pN3** | 32 (8) | 4 (12.5) | 28 (87.5) |  |  |
| **Lymphovascular invasion** |  |  |  |  |  |
| **Present** | 144 (54) | 19 (13) | 125 (87) | 0.002 | 0.96 |
| **Absent** | 123 (46) | 16 (13) | 107 (87) |  |  |
| **ER status** |  |  |  |  |  |
| **Positive** | 274 (67) | 36 (13) | 238 (87) | 2.476 | 0.12 |
| **Negative** | 132 (33) | 26 (20) | 106 (80) |  |  |

*Table continues*

**Supplementary Table 1. (cont’d)**

| **Variable** | **Total** | **Dicer positive**  **(Intensity 2, 3)** | **Dicer negative**  **(Intensity 0, 1)** | **χ^2^** | **p value^b^** |
| --- | --- | --- | --- | --- | --- |
|  | **n^a^(%)** | **n^a^(%)** | **n^a^ (%)** |  |  |
| **PR status** |  |  |  |  |  |
| **Positive** | 236 (57) | 35 (15) | 201 (85) | 0.05 | 0.82 |
| **Negative** | 180 (43) | 29 (16) | 151 (84) |  |  |
| **HER2** |  |  |  |  |  |
| **Positive** | 64 (14) | 16 (25) | 48 (75) | 4.29 | 0.04 |
| **Negative** | 380 (86) | 53 (14) | 327 (86) |  |  |
| **Triple negative** |  |  |  |  |  |
| **Yes** | 57 (14) | 11 (19) | 46 (81) | 0.5 | 0.48 |
| **No** | 355 (86) | 52 (15) | 303 (85) |  |  |
| **Molecular subtype** |  |  |  |  |  |
| **Luminal A** | 277 (76) | 34(12) | 243 (88) |  |  |
| **Luminal B** | 27 (7) | 4 (15) | 23 (85) | 0.006 | 0.94^d^ |
| **HER2 overexpressing** | 34 (9) | 11 (32) | 23 (68) | 8.31 | 0.004^d^ |
| **Basal-like** | 27 (7) | 5 (19) | 21 (81) | 0.50 | 0.48^d^ |
| **Ki67 labelling index** |  |  |  |  |  |
| **<10%** | 294 (71) | 43 (15) | 251 (85) | 0.02 | 0.89 |
| **≥10%** | 119 (29) | 18 (15) | 101 (85) |  |  |
| **BCL2** |  |  |  |  |  |
| **Positive** | 226 (55) | 26 (12) | 200 (88) | 3.24 | 0.07 |
| **Negative** | 186 (45) | 34 (18) | 152 (82) |  |  |
| **CK14** |  |  |  |  |  |
| **Positive** | 80 (21) | 18 (22.5) | 62 (77.5) | 3.29 | 0.07 |
| **Negative** | 297(79) | 40 (13) | 257 (87) |  |  |
| **CK5/6** |  |  |  |  |  |
| **Positive** | 52 (14) | 8 (15) | 44 (85) | 0.03 | 0.87 |
| **Negative** | 326 (86) | 53 (16) | 273 (84) |  |  |
| **EGFR** |  |  |  |  |  |
| **Positive** | 54 (13) | 14 (26) | 40 (74) | 4.73 | 0.03 |
| **Negative** | 356 (87) | 48 (13) | 308 (87) |  |  |
| **p53** |  |  |  |  |  |
| **Positive** | 83 (21) | 15 (18) | 68 (82) | 0.52 | 0.47 |
| **Negative** | 318 (79) | 45 (14) | 273 (85) |  |  |

a, number of cases for which Dicer expression and data for the relevant parameter was available

b, p values < 0.0025 were considered significant when the Bonferroni correction for multiple tests was applied.

c, UICC TNM Classification of Malignant Tumours 7^th^ Edition [70]

d, compared to luminal A subtype.
